# The Influence of Connection on Early Career Nurses' Rural Experiences: A Descriptive Phenomenological Study

**DOI:** 10.1155/2024/8867213

**Published:** 2024-03-06

**Authors:** Rebecca Barry, Latitia Kernaghan, Elyce Green, Claire Ellen Seaman

**Affiliations:** Locked Bag 588, Three Rivers Department of Rural Health, Charles Sturt University, Tooma Way, Wagga Wagga, NSW 2678, Australia

## Abstract

**Introduction:**

Rural nursing careers offer a multitude of benefits for individuals. Despite this, there continues to be a growing deficit in the number of nurses choosing to practice in rural areas. As the first 12–18 months of a nursing career are fundamental in shaping career location decisions, it is important to explore factors that influence early career nurses' employment decisions.

**Methods:**

A phenomenological study was undertaken to explore early career nurses' experiences during their first year of rural practice and describe how the nurses' experiences influenced their decision to remain in rural employment. Data were collected via semistructured interviews and underwent inductive thematic analysis.

**Results:**

Seven early career nurses practicing in rural locations were interviewed and described several influences on their career location decisions, particularly related to whether they would stay in or leave their rural employer. The themes derived from the nurses' stories included the effect of their vulnerability entering a new workplace, the importance of connection to person, place, and profession and the nuances of rural nursing rhythms. These had implications on their employment decisions.

**Conclusion:**

This research demonstrates the distinct form of nursing practice that occurs in rural areas which was experienced by the early career nurses as a breadth of skills, volume of presentations, and continuity of care. The nurses described the importance of establishing connections to person, place, and their profession. These connections can support nurses through a period of vulnerability entering a new workplace.

## 1. Introduction

Rural nursing careers can provide a multitude of benefits for individuals, including higher wages, providing a sense of belonging, and allowing nurses to work to their full scope and develop generalist nursing skills [1]. Despite these benefits, there remains a significant shortage of nurses working in rural areas, which is expected to continue to grow [2, 3] despite an increasing number of graduates [4]. The rural nurse shortage has led to significant efforts to conceptualise and explore ways to retain staff in these locations. The first two years of a nurse's career are important in this endeavour, as the first 12–18 months of employment has been reported as the period in which a decision is made to stay or leave. To date, research that has explored the experiences of early career nurses has largely been conducted in metropolitan settings [5–9]. Rural nurses have a distinct scope of practice which includes functioning as an advanced generalist, being intricately linked with community, and holding significant responsibilities in delivering health services [10, 11]. It is therefore important to conduct research that considers practice from the unique perspective of rural nurses. There has been research conducted on recruitment and retention specifically in rural areas, but this has not included early career nurses as a sample group [2].

This qualitative study focuses on the experiences of early career nurses practicing in rural areas in New South Wales (NSW), Australia, and describes the influences on their retention. Early career nurses in this context refer to nurses registered with the Australian Nursing and Midwifery Board, which requires them to have completed a three-year undergraduate degree consisting of theoretical and practice-based education. Many early career nurses employed in the NSW public health system are enrolled into a program that focuses on the first year of transition to practice. Programs similar to this have been evaluated previously and found to be highly variable in terms of content and success [12]. There is also evidence that the effect of factors such as ward culture and experiencing a sense of social inclusion influence the retention of junior nurses, although this remains largely underexplored in research [13–15]. The purpose of undertaking this research was therefore to contribute to an increased understanding of the influences on early career nurses' employment decisions in rural areas.

To do this, a descriptive phenomenological design was used to privilege the experiences of the early career nurses, understanding they are unique and highly impactful on their employment decisions. Descriptive phenomenology (versus interpretive phenomenology) was chosen to guide this research as it requires the researcher to use bracketing and develop an outlook of the phenomena from which they are distanced [16]. The researchers felt that this reflected the nature of the inquiry related to a phenomenon from which they are distanced (in relation to geography, discipline, and career stage) and seeking to explore broadly. This approach is appropriate for addressing the study aims as it recognises that career decision-making is complex and requires an open exploration of the reasons nurses leave their employment, as well as those that influence them to stay [17].

### 1.1. Aims

1. To explore early career nurses' experiences during their first year of rural practice.
2. To describe how the nurses' experiences influenced their decision to remain in rural employment.

## 2. Methods

This paper presents results from a qualitative analysis of interviews undertaken as part of a larger, mixed methods research project. Descriptive phenomenology was used as the aims were focused on exploring the subjective experience of early career nurses. Using this methodological approach, the researchers sought to explore how the first year of practice (the phenomenon of interest) was viewed by the individual. Ontologically and epistemologically, this research adopted a lifeworld approach based on the work of Husserl [18]. This view negotiates between subjectivism and objectivism and focuses on the relationship between participants and the world. “The lifeworld is the lived and experienced world and thus it is something more than the world itself, and more than the subject itself” [19]. The researchers involved in collecting and analysing data were guided by the methodological principles of descriptive phenomenology suggested by Sundler et al. [20], emphasising openness, questioning preunderstanding, and adopting a reflective attitude. Bracketing was conducted within the stage of openness, whereby the researchers identified and set aside their assumptions and presuppositions in order to be open to the data [16]. This research is reported in line with the consolidated criteria for reporting qualitative research (COREQ) [21] to enhance transparency and trustworthiness of the research process and results [22]. For the purpose of this study and the discussion in this paper, rural refers to locations rated 1–4 (Inner Regional to Very Remote) using the Australian Statistical Geography Standard (ASGS) Remoteness Structure [23].

### 2.1. Participants and Data Collection

Participants were purposively recruited from orientation sessions for cohorts of early career nurses (first year of practice) commencing in either of two rural local health districts in New South Wales, Australia, in 2019 and 2020. Recruitment was conducted 10–12 months from when the nurses commenced working in their role to capture them at a stage where they had experience of the phenomenon (working as an early career nurse) and were still located in their rural site (the early career program is for 12 months, and many nurses relocate after this period). The total size of the cohort was 175 nurses who were entering the workforce having become a registered nurse within the previous 6 months. Prospective participants were able to identify interest in being interviewed by noting their contact details at the end of a survey that was administered during orientation sessions as part of the larger research project. Coercion bias was mitigated by using a research team who were not known to the nurses and employed by a separate organisation. Outcomes of the research were not tied to the nurses' employment. Nurses who left their contact details were contacted by a researcher (EG or RB) to discuss the project and then emailed the information and consent sheets. After one week, the researcher invited the participant to an interview, arranged at a time of convenience for the participant.

Interviews were conducted by two members of the research team (EG and RB) and were completed virtually using an online videoconferencing platform. Interviews took between 27 and 46 minutes (with a mean time of 36 minutes). A semistructured interview guide was used to conduct the interviews (Table1) The interview guide was developed based on the information required to answer the research question and concepts from the literature thought to affect the experiences of early career nurses (i.e., social connections, career ambitions, and placement locations). One question was added to the interview guide following the second interview as the participant raised the impact of COVID-19 as being significant for their experience as a nurse, so this was asked of subsequent participants. Data saturation was not used as guidance for participant numbers, taking the view of Braun and Clarke [24] that meaning is generated through the analysis rather than reaching a point of saturation. The sample size was therefore guided by how many nurses opted in to the research and a sample of seven is sufficient considering the phenomenological approach [25, 26]. For all interviews, only the researcher and the participant were present and each interview was video-recorded and then transcribed verbatim. Written informed consent was obtained from each participant prior to the interview, and no participants withdrew after providing consent. Each interviewee was offered to be sent a copy of the interview transcript to review and make any comments, explanations, or amendments; however, none opted to do so.

### 2.2. Data Analysis

Thematic analysis of interview transcripts was undertaken using a descriptive phenomenological approach. Guidance from the work of Sundler et al. [20] was used, whereby the process of analysis incorporates visiting the interview transcripts as a whole and as individual descriptions of the phenomena, with the researchers moving between closeness and distance from the data. Data analysis was conducted by three members of the research team (RB, LK, and EG). The three researchers who undertook data analysis identify as women and are from the disciplinary backgrounds of social work, occupational therapy, and nursing, respectively. The three researchers all work at a regional university and are involved in programs that focus on the recruitment and retention of rural health professionals. As health professionals, they have all practised in rural Australia for >10 years.

During analysis, each researcher considered their preunderstanding of the phenomena and conducted open-minded reading (bracketing) before exploring the meanings embedded in each interview. The process of data analysis was highly reflective, and the team met frequently throughout the process of analysis to discuss and reflect on results. Thematic analysis was inductive, and the themes presented in the results of the research were data-driven. To reflect the importance of the individual story for understanding the results of the research, each participant was given a pseudonym name and their story presented as part of the results. This acknowledges the different journeys and experiences of each person. Quotations used in the results section aim to give voice to each participant and are attributed to the person from whom the words came.

### 2.3. Ethical Approval

This research was granted human research ethics approval by the Greater Western Human Research Ethics Committee, approval number 2019/ETH00108.

## 3. Results

### 3.1. Summary of Participants

Seven early career nurses, all of whom identified as women and had been working as a registered nurse for 10–12 months, opted to be interviewed for this research. A summary of the participants and their stories is presented in Table 2.

### 3.2. Thematic Analysis

A descriptive phenomenological approach was undertaken to analyse the data, and themes were inductively developed using the experiences of the participants. There were three main themes identified in the data that related to the individual experiences of the participants and told a story about the overall influences on the nurses as they entered professional practice and subsequently made decisions about employment. The themes included “The effect of vulnerability during a period of transition,” “Connection to person, place, and profession,” and “Nuances of rural nursing rhythms” and are presented below with reference to the experiences of the individuals.

### 3.3. The Effect of Vulnerability during a Period of Transition

The theme “Effect of vulnerability during a period of transition” demonstrates the experiences of the early career nurses joining a new workplace and, in many cases, a new profession. During this transition time, many of the nurses spoke of their feelings of vulnerability, and how this resulted in their experiences having a heightened effect on them. Riley's experience demonstrated this

“…it was a big shock to my system. My previous job was 9 to 3 Monday to Friday and now I'm full-time on a rotating roster. So trying to balance social life and adulthood with work especially having to drive ….” (Riley)

Similarly, Jesse felt that the transition had a significant effect on her familial and social connections.

“I think for the first couple of months I didn't see anyone or talk to anyone. I was just too tired, and too overwhelmed. And there was so much new knowledge coming at me all the time that I–it was just–it was a good effort if I could do the dishes on that day, or make my bed. There was–it was hard. Now it's a little bit better.” (Jesse)

The vulnerability felt by the nurses resulted in their initial experiences being strongly influential on their happiness and decisions to remain or leave. Sam described receiving a warning when she first arrived in the new town and how this affected her entire experience.

“So, when I started, I remember the first day, my NUM (nurse unit manager) came to pick me up … and the first thing she said to me that really surprised me was, she said we've got a lot of strong characters here and I was like okay and I didn't know what it means” (Sam)

For Sam, this conversation put her on alert for conflict and affected the way she approached the role. In contrast, some participants spoke to the value of a supportive, welcoming, and positive atmosphere within the team where skill development, feedback, and teamwork were modelled by existing team members. Examples of this included being supernumerary for two weeks, monthly debriefing session, and creating and extending their social connection to the community, as was demonstrated by Alex.

“In the country hospitals, the senior nurses are really lovely, and they'll always back you up, and then if you're not unsure what's happening with a patient, they'll lead you and they'll tell you where you need to look, and what information you can investigate or research or do more studying. So, you'll feel like a real nurse then…” (Alex)

The impact of a transition period of vulnerability for many of the nurses meant that their initial introduction into the rural workplace had lasting and significant effects. The initial positive or negative experiences influenced their career choices. One exemplification of this was described by Sam who had applied for a rural nursing position and was excited to commence a rural career. The negative work culture and limited support experienced by Sam led her to relocate to a different rural facility at the end of her one-year contract.

The research participants did describe some protective factors that assisted them during their period of vulnerability and negated some of the effects of their experiences. These were described as having previously undertaken shift work, having lived locally, or having skills that were transferable to this new environment. An example of this was shared by Kelly.

“I know like a lot of people say like time management's really difficult in your first year out, but, for me, because I had already been an enrolled nurse and I think, you know, I'm a mum of, you know, 4 kids and so I feel like, for me, my time management skills are pretty spot on because I've had to be able to study and be able to work.” (Kelly)

In summary, this theme demonstrates the increased vulnerability of early career nurses in relation to personal and professional challenges, the influence this has in terms of heightening the effect of positive and negative experiences, and some of the skills and experiences that can potentially mitigate some of these effects.

### 3.4. Connection to Person, Place, and Profession

The early career nurses who participated in this research felt their experiences had been moulded through the connections they made to people, place, and the profession. This was largely spoken of in the context of working in a rural workplace. Feelings of belonging in their new environment were important to the nurses. This was shown by Kelly who described,

“… we know a lot about each other, we share a lot of information about ourselves and our family because we work really long hours [together]… these people that we work with become your extended family, and that's definitely a rural [hospital].” (Kelly)

Many of the participants described the positive effect of colleagues being open and happy to share knowledge, creating regular social catch ups either inside or during work hours (i.e., regular lunch outside of the hospital). Other examples were colleagues attending the same Church, organised group nights out, text messaging after work to check in, sharing of food in the staff room, and invitations to colleagues' homes.

Feelings of belonging were not discussed in the isolated environment of the workplace. Two of the nurses who were established in the rural location prior to their new career felt a sense of belonging that extended into the community. Kelly explained,

“… Most of the other nurses and colleagues I work with know a lot about my family and they will see my children down the street or at the shops and most of them, you know, my children, you know, share a special bond with them as well and – yeah. So that's probably a really good thing about working rurally…” (Kelly)

Darcy also discussed connection and belonging in relation to the wider rural community,

“…I usually can relate to most people because I'm married to a farmer as well. So a lot of the patients have got farming–are either farmers themselves, or married to farmers, or connections to–like you sort of feel connected more than just one person–on a personal level. You sort of feel like you're connected I guess locationally as well …” (Darcy)

As a profession, nursing also connected the participants to the community. Darcy described how the nursing uniform connects people “because nearly everyone knows someone that works at the hospital.” Kelly had similar sentiments about the nursing connection to community and spoke about knowing patients as people,

“I like to work in rural areas because I'm in a community where you see these people that we discharge and we see them down the–you know, at the supermarket or we see them somewhere out and they recognise us and we recognise them and in some ways you follow up with their story … the whole story and their journey that they go through with their health …” (Kelly)

Although feeling connected to the rural community influenced the nurses' decisions to stay working in this location, feeling disconnected had the opposite outcome. Sam described feeling disconnected which led her to relocate to a different rural town. Reflecting on her experience, Sam explained

“I think it was not culturally, it's not, it wasn't, I wasn't, I didn't feel like I was part of it. Like after work I had no life and you know, I tried going out exercising and all that kind of stuff, but you know, when the nurses, they don't involve you in activities…” (Sam)

For Sam, the segregation had significant effects and she got to the point where

“I don't think I can survive here … you would finish work after a stressful shift and all you need is a friend and you've got no one there. There is no one…” (Sam)

This theme demonstrates the nurses' experiences of connection and how this influenced their career decisions. These findings show that nurse retention is influenced by factors not solely related to their learning and clinical practice but is connected with their personal embeddedness in a community. Riley explained

“… You just feel so loved and supported which is going to be hard to say goodbye to if I do decide to go onto somewhere else.” (Riley)

### 3.5. Nuances of Rural Nursing Rhythms

The participants described their experiences of rural nursing and discussed this type of work as being a distinct form of practice. They highlighted that, in rural areas, patients are often significant people in their community and/or personal life. Participants described the rhythm of rural practice as being unique as they often followed someone through their entire admission, working across specialties from emergency to aged care. Kelly explained

“I really enjoy seeing someone's life–I suppose–like the whole story and their journey that they go through with their health and being able to try to help them in those ways.” (Kelly)

The practice scope of rural nursing and this continuity across a person's health journey was also described as facilitating greater rapport and understanding of a person's individual health and circumstances. Sam even described one experience of closeness with a patient

“Being there for a patient like that when there is no family member, you know, holding his hand, just telling them it's okay I'm here with you. As a graduate nurse, that was the beauty I had there was, you know, you do everything…” (Sam)

Rural nursing rhythms were also described in relation to the volume of emergency presentations which, in some sites, were infrequent but challenging due to the variety and unpredictability. This required a unique skill set particularly when working without other health professionals onsite in a geographically isolated area. This perspective contributed to the narrative of rural nursing as a distinct form of practice requiring a broad set of skills.

In addition to the professional considerations of a rural nursing career, Jesse described the challenges it could pose when negotiating shift work and personal commitments,

“… I don't feel I have enough time off to go and see my family. It's a weekend trip if I go, it's 3 hours, or 5 hours depending on which I go. And so I think yeah having 2 days off after you finish on an afternoon is–it's too hard to make an effort to go to–to go either–either place. So I haven't seen them in a long time.” (Jesse)

These professional considerations therefore also interact with connections and, in this instance, affected the nurse's access to support networks. Although this was a complication for Jesse, for Alex who had also newly moved to the area, the rural rhythm was one of contentment.

“… you have your own peace and quiet, and then if you want that social life, you can have social life, you can go to see other people, or you can organise something” (Alex)

This theme demonstrates the early career nurses' description of rural nursing rhythms as being a distinct form of practice, allowing for continuity of care, and being considered in the context of geography. The nurses reflected positively on their clinical experiences, and there were no instances of the nurses reporting an intention to leave based on clinical experiences.

## 4. Discussion

Early career nurses have been recognised as a cohort of nurses who can be highly mobile due to a range of personal and professional factors [27]. This research sought to explore the experiences of early career nurses in rural areas to determine the influences on the likelihood they would be retained in their rural workplace. It was identified that during the period of transition into the workplace, these nurses were vulnerable to the effects of their environment, such as their work culture and connectedness to the rural community. This heightened vulnerability may, in part, be explained by a phenomenon known as culture shock [28] or transition shock [29], caused by a misalignment between expectations and reality of entering the nursing workforce.

The vulnerability of early career nurses during the transition into the workplace can put them at risk of negative outcomes associated with poor workplace culture. Clarke et al. [30] previously estimated that 60% of nurses will leave their first job due to the behaviours of their colleagues. Our research demonstrated that for Sam, the segregation from colleagues was the primary reason for leaving her employment. Sam spoke about experiences of feeling like an outsider and the way that this influenced her life within and outside of work. In contrast, other nurses had experienced feelings of being “part of an extended family.” These experiences of the early career nurses as insiders versus outsiders reflect similar findings of research conducted by Ho et al. [31]. They described how early career nurses experienced “support and belonging” or “feeling unsupported and alienated” [31]. Explored through the lens of job embeddedness, Ho et al. [31] found that early career nurses who felt like outsiders were less likely to be retained in the workplace. Considered in relation to a rural context, it is potentially even more important to connect early career nurses to their workplace and colleagues, especially those new to the area or without strong existing connections who are more likely to be socially and geographically isolated.

In the context of early career nurse experiences and retention, engaging in socialisation tactics may be valuable for rural health organisations as they attempt to support newcomer nurses transitioning into the rural health workforce [32]. These tactics include various mechanisms focused on transforming “an outsider to an effective insider” [32] and supporting an employee to adapt to the new environment, behaviours, and knowledge. Suggested mechanisms to achieve this in nursing include structured rotations, staff support, ongoing education, and mentorship programs [6, 33, 34]. Research by Saks et al. [35] has demonstrated the relationship between socialisation tactics and newcomer adjustment, finding tactics that represent institutionalised socialisation were negatively related to factors such as intentions to quit and positively related to factors such as job satisfaction, performance, and organisational commitment. In the rural environment, further consideration should also be given to interplay between collective and individual socialisation due to the size of the population. Results from this study suggest that connections with colleagues may be of greater significance in rural areas due to the limited interpersonal interactions that occur outside of work, particularly for a newcomer to the community. Outside of socialisation tactics, our research also showed that some of the early career nurses felt that prior experiences such as having previously done shift work, having lived locally, or having skills that were transferable to this new environment acted as protective factors during their transition phase. These factors could be further explored in future research.

The implications of feelings of connectedness in rural nursing practice have previously been discussed by Conger and Plager [36]. They found that connectedness was essential for the retention of rural nurses and “graduates who reported a sense of disconnectedness when working in a rural community were less likely to remain in that community” [36]. An additional consideration discussed by Conger and Plager [36] was the experience of connectedness for rural nurses entering a new community. For nurses entering rural communities as outsiders, there is added complexity in establishing connections as they are starting with minimal ties. The imperative to establish connections is significant due to its influence on both recruitment and retention of rural nurses [36]. This reflects the experience of several of the early career nurses discussed in our research as the rural origin nurses described maintaining connections as a protective factor, and those moving in from outside the communities described the connection as a primary influence on retention.

In research focused on understanding flourishing among rural nurses, Crawford [37] also identifies connection as important and that “rural nurse participants felt a deep connection to their communities and their role within the community as a nurse.” In this context, Crawford [37] used Edgar and Pattinson's [38] definition of flourishing, which they relate to wellbeing, but point out that it additionally captures vulnerability and suffering to “create a framework through which one can meaningfully and constructively go on with one's life” (p. 161). Crawford found that “working with a purpose” helped rural nurses flourish [37]. In our research, the early career nurses discussed being able to work with people who were important to them and/or a part of their community, as well as being able to provide a continuity of care and see the outcomes of their work. This was very similar to the activities attributed by Crawford as contributing to a sense of purpose and flourishing in rural nursing, including working across diverse role and skills, and the meaning behind each encounter with a patient [37].

Connection therefore plays an important role in shaping the experiences of rural nurses. Although the results of our research demonstrated that this role was largely positive, other research studies focused on rural nurses has demonstrated that connection to communities can create difficulties for some nurses when personal and professional boundaries become blurred [39, 40]. The experience of early career nurses in navigating professional and personal boundaries is worthy of future exploration.

Rural nursing has been acknowledged as a distinctly unique form of practice for more than 20 years [28, 41, 42]. It is therefore unsurprising that the early career nurses in this study identified it as such. Interestingly, the early career nurses did not reflect on their clinical practice as a significantly influential factor in their career decisions. They instead pointed to factors such as their connections with people, place, and profession as being primarily related to their employment location. This is different to the findings of similar research conducted by Rose et al. [33] who also used a phenomenological approach to explore the experiences of early career nurses in rural areas. Rose et al. [33] found several factors that influenced nurses' experiences, including professional factors such as scope of practice, need for ongoing education, specialty rotations, and lack of staff and resources. They did, however, also find several personal factors that influenced the early career nurse experience, including the sense of community, importance of collegial support, and maintaining a work/life balance [33]. These differences in findings may be attributed to a difference in years of experience of the nurses recruited for Rose et al. [33] research as compared to our research. As several (10 out of 13) nurses interviewed by Rose et al. [33] had been practicing for >1 year, it is possible the time they had to adjust to their career and location may have changed the emphasis they placed on different factors that influenced their career decisions. The nurses in our research had all been practicing for ≤1 year and therefore had less time to establish personal and professional connections, thus placing these at the fore in their experiences.

This study has highlighted that connectedness and inclusion in both the workplace and the community for nurses during the period of transition cannot be underestimated and can impact on retention in rural areas. These findings have implications for employers and communities supporting nurses and emphasises the importance of prioritising, planning, and implementing practical strategies to promote inclusion, connectedness, and a positive workplace culture. Strategic and specific ways in which to assist early career nurses to experience a sense of belonging and embeddedness in the workplace and transition to the unique experience of rural nursing present an opportunity for future research.

There are a number of limitations inherent to the design of this study. Participants were recruited via their workplace and thus although steps were taken to ensure they understood the research would not affect their employment, it is possible some nurses perceived an association between the research and their employment. Asking participants to self-select participation in the project may also have resulted in nurses who had either very positive or very negative experiences opting to participate, as they may be more likely to want to discuss their experiences. Finally, as this research was exploratory, it is not generalisable to the broader population but instead provides a platform for further research on this topic.

## 5. Conclusion

This study demonstrates the vulnerability felt by early career nurses as they transition into a new profession and how this can result in a heightened response to experiences during the initial 12 months of practice. The nurses in this study described the distinctness of rural nursing as a profession, and the benefits associated with this type of practice, such as using a variety of skills and providing care, cross a continuum. Primarily, the nurses' career decisions were influenced by their experience of connection to person, place, and profession. These results can be used to further conceptualise the importance of connection and belonging in achieving retention of staff in rural areas.

## Figures and Tables

**Table 1:** Semistructured interview guide.

	Question	Additional questions to probe for more information
1	Tell me about why you chose to become a nurse?	*(i) Probing for life experience or influences on career choice*
	What kind of nurse do you see yourself as?	*(ii) Looking for an understanding of if/how they identify themselves as rural or working within a rural context*

2	Tell me about how you decided to come and do the transition to professional practice in this local health district?	*(i) Probe for other experiences of living/working in rural areas before study, during study etc.*
		*(ii) Feelings of connection to the area*

3	During your undergraduate studies for nursing, did you do any clinical placements in a rural/regional/remote area?	*(i) Tell me about your experience/s*

4	Did these experiences affect your decision to take a position in this local health district?	*(i) What influenced your decision-making?*

5	What were the best and most challenging parts of being a new graduate?	*(i) What do you think are the best parts about being a new graduate?*
		*(ii) Le*t*'s talk about the most challenging parts of being a new graduate…*

6	Tell me about your experience of COVID-19 and the effect it has had on you (if any) personally and professionally?	*(i) Looking at the effects of the pandemic on both social and professional experiences*
		*(ii) Has your perspective of your future career as a nurse changed due to COVID?*

7	Tell me about the social, nonwork side of things over the past year while you have been doing the program?	*(i) Social and community connections, keeping in touch with family and friends, feeling a sense of belonging*

8	What are the things that would/have made you want to stay?	

9	What do you think the future holds for you career-wise?	*(i) Looking to see what opportunities they perceive are available, factors that affect decision to stay or leave and where they will be seeking future employment*
		*(ii) What's next for you?*

**Table 2:** Summary of participants and their stories.

Summary of participants' experiences
*Sam's story*:
(i) Applied for new graduate program in rural area as heard positive stories from other student nurses about rural nursing, no family living in the area
(ii) Experienced “negative” work culture and limited clinical support. Did not experience a sense of belonging, inclusion, and connection at work and in the community
Outcome: relocated from the area where the new graduate program was completed and secured employment in an outer regional area

*Alex's story*:
(i) Applied for new graduate program in rural area for experience and employment prospects, no family living in the rural area
(ii) Felt supported by senior nurses and team. Included and connected to people outside of work
Outcome: relocated back to major city area after completing the new graduate year to be with family

*Darcy's story*:
(i) Applied for new graduate program in rural area as previously worked as an enrolled nurse there. Strong connection to the rural area and rural people. Family living in the area
(ii) Some feelings of being overwhelmed by the clinical work as a new graduate. Felt welcomed into the team and well supported
Outcome: despite an interest in relocating to work in a larger hospital, decided to stay in the rural area and consider relocating after 5 to 10 years

*Charlie's story*:
(i) Applied for new graduate program in rural area as already residing there. Strong connection to the rural area and rural people. Family living in the area
(ii) Some challenges related to the COVID impact on work, including reduced access to study days and professional development. Felt welcomed into the team and well supported
Outcome: stayed in the rural area after new graduate year as felt familiar with the service and seeking job security

*Kelly's story*:
(i) Applied for new graduate program as worked as an enrolled nurse there and felt supported by management. Strong connection to the area and rural people. Family living in the area
(ii) Some challenges related to the COVID impact on work, such as access to professional development. Felt supported at the service, attracted to the familiarity and flexibility of the service
Outcome: stayed working in the rural area after new graduate year for career and professional development opportunities, support from colleagues and the flexibility with family commitments

*Riley's story*:
(i) Applied for new graduate year in Sydney and Melbourne as intended to move to city. Accepted new graduate offer in a rural area after being unsuccessful with first preference
(ii) Some challenges related to balancing shift work and social life. Felt welcome and supported, developed connections with colleagues and community. Enjoyed learning exposure, opportunity to be in charge
Outcome: stayed in the rural area after the new graduate year for financial, social, and lifestyle reasons

*Jesse's story*:
(i) Applied for new graduate year in rural area as already residing there and hesitant to leave, family close by, strong connection to rural areas and people
(ii) Some challenges related to the COVID impact on work, adjusting to full time work and making connections with people due to age. Felt a sense of belonging and experienced a positive culture at work
Outcome: planned to stay in the rural area after new graduate year for support, connection with colleagues and community

## Data Availability

Due to the ethical obligations of the authors, the data used in this study are not available.

## Supplementary Materials

Supplementary Materials The COREQ checklist is included as additional supplementary information to detail the location of pertinent data within the manuscript.
